# *Campylobacter* in Broiler Chicken and Broiler Meat in Sri Lanka: Influence of Semi-Automated vs. Wet Market Processing on *Campylobacter* Contamination of Broiler Neck Skin Samples

**DOI:** 10.3390/foods6120105

**Published:** 2017-11-29

**Authors:** Kottawattage S. A. Kottawatta, Marcel A. P. Van Bergen, Preeni Abeynayake, Jaap A. Wagenaar, Kees T. Veldman, Ruwani S. Kalupahana

**Affiliations:** 1 Department of Veterinary Public Health and Pharmacology, Faculty of Veterinary Medicine and Animal Science, University of Peradeniya, Peradeniya 20400, Sri Lanka; sarunika@yahoo.com (K.S.A.K.); preeniab@gmail.com (P.A.); 2 Wageningen Bioveterinary Research, 8221 RA Lelystad, The Netherlands; Marcel.vanBergen@radboudumc.nl (M.A.P.V.B.); J.wagenaar@uu.nl (J.A.W.); Kees.veldman@wur.nl (K.T.V.); 3 Department of Infectious Diseases and Immunology, Faculty of Veterinary Medicine, Utrecht University, 3508 TD Utrecht, The Netherlands; 4 WHO Collaborating Center for Campylobacter/OIE Reference Laboratory for Campylobacteriosis, 3508 TD Utrecht, The Netherlands

**Keywords:** campylobacter, broiler chicken, poultry processing, Sri Lanka

## Abstract

Broiler meat can become contaminated with *Campylobacter* of intestinal origin during processing. The present study aimed to identify the prevalence of *Campylobacter* in broiler flocks and meat contamination at retail shops, and determine the influence of semi-automated and wet market processing on *Campylobacter* contamination of neck skin samples. Samples were collected from semi-automated plants (*n =* 102) and wet markets (*n =* 25). From each batch of broilers, pooled caecal samples and neck skin samples were tested for *Campylobacter*. Broiler meat purchased from retail outlets (*n =* 37) was also tested. The prevalence of *Campylobacter* colonized broiler flocks was 67%. The contamination of meat at retail was 59%. Both semi-automated and wet market processing resulted to contaminate the broiler neck skins to the levels of 27.4% and 48%, respectively. When *Campylobacter*-free broiler flocks were processed in semi-automated facilities 15% (5/33) of neck skin samples became contaminated by the end of processing whereas 25% (2/8) became contaminated after wet market processing. Characterization of isolates revealed a higher proportion of *C. coli* compared to *C. jejuni*. Higher proportions of isolates were resistant to important antimicrobials. This study shows the importance of *Campylobacter* in poultry industry in Sri Lanka and the need for controlling antimicrobial resistance.

## 1. Introduction

Campylobacteriosis is a major public health concern worldwide. Out of the 27 *Campylobacter* species and eight subspecies identified so far, *Campylobacter jejuni* (*C. jejuni*) and *Campylobacter coli* (*C. coli*) are the foremost two species causing campylobacteriosis in humans [1,2]. Even at a low infectious dose of only a few hundred bacteria there is a reasonable chance that an infection will be established in humans [3,4]. *Campylobacter* has developed resistance to several antimicrobials, in particular, resistance to fluoroquinolones [5]. A recent study conducted in Luxembourg found that around 61% of *Campylobacter* infections in humans were attributed to poultry meat, 33%, 5% and 0.6% were attributed to ruminant meat, the environment and pork meat, respectively [6]. Among poultry, chicken is the major animal reservoir for *Campylobacter* [7]. It has been estimated that 20–30% of the human campylobacteriosis cases can be attributed to handling, preparation and consumption of broiler meat; 50% to 80% may be attributed to the chicken reservoir as a whole [8].

*C. jejuni/coli* species colonize the gastro intestinal tracts of poultry as commensals, and hence, disease in the colonized birds is not readily apparent. Broilers that enter processing plants may carry very high numbers of Colony Forming Units (CFUs) of *Campylobacter* in their intestines, caeca, cloaca, crops and feces [9,10,11,12]. *Campylobacter* present in the gut of colonized broilers may contaminate the meat and survive as the meat passes through the processing line leading to the possibility of widespread cross contamination within the processing plant. Rinsing steps during processing may further aggravate the problem of contaminated carcasses by introducing *Campylobacter* organisms even to uncontaminated carcasses [13,14]. 

Due to the risk of contamination the effects of different variables during broiler rearing, of interventions during processing and of different processing steps on the level of meat contamination by *Campylobacter* have all been studied [15,16,17]. However, research that has directly compared automated and wet market processing on the levels of meat contamination with *Campylobacter* is sparse. This could be due to the fact that the majority of the studies have been carried out in industrialized countries where automated processing is the common practice. 

According to the Food and Agriculture Organization (FAO) the majority of large, medium and small scale poultry processing operations in most parts of the globe are generally fully automated [18], but this is not the case in many Asian countries, where other methods of poultry processing are still in operation. For example in India, approximately 90% of broiler meat is still manually processed [19]. 

Poultry is the leading livestock industry in Sri Lanka and chicken is the only meat type exported [20]; understanding the extent and causes of *Campylobacter* contamination is therefore important for human health and economic welfare. Poultry meat remains the most commonly consumed meat type [21]. In Sri Lanka, broiler meat is largely produced in large scale or semi-automated processing plants [20,22]. Large scale processing plants in Sri Lanka [20,22] practise standard methods such as: pre-processing, slaughter, evisceration, secondary processing, packing and shipping [23]. However, vent opening and evisceration are carried out manually; thus, the term semi-automated is used in the present study. Usually each slaughter batch at a semi-automated facility consists of 1000 birds or more. Small-scale, or wet market processors, on the other hand slaughter birds depending on the day to day requirement and mostly process less than 100 birds. Generally, a few cages of live birds are kept on the premises of wet market processors. In these facilities both slaughtering and making the bird ready for sale are usually carried out in one small area and on the same work surface. 

The objectives of the current study were to (i) investigate the prevalence of *Campylobacter* in Sri Lankan broiler chicken, (ii) to investigate the influence of processing methods and (iii) to determine antimicrobial resistance profiles of the isolated strains. 

## 2. Materials and Methods

### 2.1. Sample Collection

During the period of October 2006 to July 2007 caeca contents and neck skin samples were collected from 127 flocks of broilers during processing in two semi-automated (plants A and B) and four wet market processing facilities. The caeca samples were collected at the point of evisceration and the neck skin samples were collected after washing with chilled water. 

In semi-automated processing plants two pooled samples from each flock were obtained by mixing the caecal contents of five birds. Both samples were analyzed for *Campylobacter*, if one or both of the samples tested positive the flock was considered as positive. From each flock one pooled sample of neck skin was obtained by blending ten neck skins.

At wet markets caeca and neck skin samples were obtained by pooling material from 1–5 animals, depending on the availability.

Unprocessed broiler meat samples for human consumption were purchased from 37 different retail shops. All samples were transported in a cool box and analyzed immediately. It was not possible to identify the method of processing. 

### 2.2. Isolation and Identification of Campylobacter

Identification was performed following the method given by ISO 10272:1995(E) with certain modifications. Samples from caecal material were directly cultured on modified Charcoal Cefoperazone Deoxycholate agar (mCCDA) plates. Twenty five grams from the pooled neck skins or retail meat samples were enriched in 225 mL of Preston enrichment broth, incubated at 42 °C for 48 h. After enrichment, a loopful taken from a wire loop was plated on mCCDA plates. The mCCDA plates were incubated at 42 °C for 24 to 48 h at a microaerobic atmosphere which was created using a Campy-gen gas pack. All the media and Campy-gen gas packs utilized were from Oxoid. 

Culture plates were observed after 24 to 48 h for the presence of typical *Campylobacter* colonies. Suspected colonies were selected and cultured on blood agar plates. Thereafter, Gram staining, catalase test, oxidase test, aerobic growth at 42 °C, anaerobic growth at 25 °C and reactions in Triple Sugar Iron (TSI) agar slants were utilized for genus identification of *Campylobacter.*

### 2.3. Species Identification and Antimicrobial Susceptibility Testing of Campylobacter

Sixty five *Campylobacter* isolates were sent to the WHO Collaborating Center for Campylobacter/OIE reference laboratory for campylobacteriosis in the Netherlands for species identification by Polymerase Chain Reaction (PCR) [24] and to the Dutch National Reference Laboratory for antimicrobial resistance in Lelystad for antimicrobial susceptibility testing. The antimicrobial susceptibility of the isolates was tested for 12 antimicrobials; erythromycin, gentamicin, streptomycin, neomycin, tetracycline, ciprofloxacin, nalidixic acid, tulathromycin, ampicillin, clarithromycin, sulphamethoxazole and chloramphenicol using the microbroth dilution technique to determine minimum inhibitory concentrations (MICs) (CSLI M31-A3, 2008).

### 2.4. Statistical Analysis

The software, SPSS was used for data analysis. 

## 3. Results

### 3.1. Prevalence of Campylobacter in Broiler Flocks and Neck Skin from Semi-Automated and Wet Markets

The presence of *Campylobacter* in caecal contents was considered as evidence that the broilers came from flocks already harboring the bacteria. In flocks processed at semi-automated plants 67.6% (69/102) tested positive for *Campylobacter* and in flocks processed at wet markets 68% (17/25) tested positive for *Campylobacter*. All broilers processed in both semi-automated plants and wet markets originated from farms practising deep litter open-house system with low-biosecurity.

In pooled neck skin samples 27.4% (28/102) were positive for *Campylobacter* at semi-automated processing facilities and 48% (12/25) were positive for *Campylobacter* at wet market processing facilities (Table 1). There was no significant difference in levels of neck skin contamination between semi-automated and wet market processing (using Fisher’s exact test, *p* = 0.0570).

### 3.2. Prevalence of Campylobacter in Broiler Meat Retail Samples

The prevalence of *Campylobacter* in chicken meat samples purchased at retail shops was 59% (22/37) (Table 1).

### 3.3. Effect of Poultry Processing Method on Campylobacter Contamination of Neck Skin

Because caecal and neck skin samples were collected from birds of the same flock the extent of cross contamination with *Campylobacter* during processing could be determined. Thirty-three *Campylobacter* free flocks were processed by semi-automated plants and 5 neck skin samples from those flocks became contaminated (15%). Eight *Campylobacter* free flocks were processed by wet markets and 2 neck skin samples became contaminated (25%). 

### 3.4. Species Identification of Campylobacter Isolates

As determined by PCR, the predominant species was *C. coli* which represent 69% of the total number of isolates tested. The tested isolates (*n =* 65) represented meat purchased from retail shops, caeca as well as neck skin collected from semi-automated plants and wet markets (Table 2). 

### 3.5. Antimicrobial Resistance Profile of Campylobacter Isolates

Antimicrobial susceptibility testing was carried out using microtiter trays (Trek Diagnostic Systems, UK) to determine the Minimum Inhibitory Concentrations (MIC) to 12 antimicrobials. The associated resistance patterns of the 65 isolates tested are shown in Table 3. Irrespective of the species, more than 80% of the isolates were resistant to ciprofloxacin and nalidixic acid. Resistance against erythromycin was 11% and 5%, respectively in *C. coli* and *C. jejuni* isolates (Table 3). 

## 4. Discussion

In order to gain an insight into the association of *Campylobacter* with the broiler industry in Sri Lanka, the prevalence of *Campylobacter* in the caeca of broiler chickens, in neck skins after processing and in meat for sale in retail shops was investigated. The effect of two poultry processing methods (semi-automated and wet market) on contamination of neck skin samples with *Campylobacter* was determined and the level of antimicrobial resistance present in the isolates was explored. 

The overall presence of *Campylobacter* in caeca collected from local broiler flocks at semi-automated and wet markets was around 67%. Apart from a previous publication from our laboratory on *Campylobacter* initial colonization in broilers and prevalence in layers at the farm level [25] this is the first study which describes *Campylobacter* in relation to the poultry industry in Sri Lanka. There are very few publications about *Campylobacter* prevalence in poultry in South Asia. Malik reported that 32% of broiler flocks were positive for *Campylobacter* by screening caecal samples collected from retail shops and slaughter plants in the Bareilly region of India [26]. The reason for the apparent higher prevalence noted in Sri Lanka may be the higher temperature in the country compared to the cooler climate of the Bareilly region of northern India. In a study of 20 broiler farms in the Lahore region of Pakistan the mean *Campylobacter* prevalence in broilers was 58%, which is in accordance with the present study [27]. Because there are few reports on flock prevalence of *Campylobacter* in South Asia the results were compared with other tropical countries. The prevalence observed in the current study is similar to that observed in Tanzania 69.8% [28] and in Brazil 80% [29]. Reunion Island, which is situated in the Indian Ocean, reported a prevalence rate of 54% in their broiler flocks [30]. A previously published cross sectional study conducted in Sri Lanka of flocks of poultry layers reported a prevalence of 64%, which is a closer value to that found in the present study [25]. In non-tropical countries, the prevalence varies strongly between countries. Based on a survey conducted by European Union, the prevalence of *Campylobacter* in broiler batches varied from 2.0% to 100.0% in European Union member state countries [31]. Besides differences in climate, differences in biosecurity may be responsible for the differences in prevalence. 

Due to the lack of clinical disease and mortality at the farm level *Campylobacter* colonization of broiler birds gets little attention; however, it is a serious matter when consumer safety is concerned. There are numerous studies describing the prevalence of *Campylobacter* contaminated broiler meat at retail shops, particularly in industrialized countries. The few reports available from Asia indicate that broiler meat contamination at retail levels is 31% in Thailand, 30% in Vietnam and 48% in Pakistan [32,33,34]. After testing 1587 fresh chicken carcasses collected from farmers’ markets and supermarkets in China, a contamination rate of 45.1% has been reported [35]. The level of contamination identified in the present study was 59%. Contamination rate of local retail meat with *Campylobacter* is slightly higher than in the other Asian studies and also to some western countries [35,36]. 

As shown in the literature, mechanical processing is less common in South Asian countries [19,37], though this situation is not the same in Sri Lanka [38]. In Sri Lanka, the majority of broiler birds are processed mechanically in semi-automated plants, but a significant number of wet markets, where only poultry is slaughtered, continue to exist. These small scale markets cater for particular groups of customers and/or certain ethnic groups. Therefore, this study attempted to compare the *Campylobacter* contamination of meat resulting from the two types of processing methods.

According to our results, the meat processed either by semi-automated processing (27.4%) or processing in wet markets (48%) resulted in neck skins contaminated with *Campylobacter*. However, direct comparison is hindered by differences in pooling samples for isolation of *Campylobacter* (five samples per pool for semi-automated and less than five for wet market samples). Further, a quantitative analysis is warranted to evaluate the actual public health risk. 

A recent study conducted in Malaysia reported that the prevalence of *Campylobacter* in chicken meat from conventional wet markets was significantly lower (70.7%) than in hypermarkets (91.4%) [39]. 

Analysis of data from the present study shows that when *Campylobacter* free flocks were processed in semi-automated facilities, there was a lower contamination (15%) than when the *Campylobacter* free flocks were processed in wet markets (25%), although this difference failed to reach statistical significance (Fisher’s exact test, *p =* 0.6059) due to the very limited number of samples. Nevertheless, a study conducted in China using a larger sample size reported 51.9% of chicken meat tested from farmer’s markets and 37.8% from supermarkets were contaminated with *Campylobacter*, but a significant difference was not observed between two. Even though the types of samples tested were different, the meat in the Chinese study, and the neck skins in the present study, there does appear to be a comparable trend [35]. 

Molecular analysis of *Campylobacter* isolates from the present study revealed a higher percentage of isolates being *C. coli* than *C. jejuni.* A similar preponderance of *C. coli* was observed in Thailand [40], but in many other countries, *C. jejuni* was the predominant species [41,42]. A study conducted by Malik and others in the Bareilly region, India [26] also found that out of the 32 *Campylobacter* isolates recovered from chicken caecal samples 30 (93.5%) were *C. coli* and 2 (6.25%) were *C. jejuni.* Studies from Pakistan reported 35.2% of isolates were *C. jejuni* from healthy broilers [43]. Another report from the same country (Pakistan) [27] stated that 88% of isolates from cloacal swabs of live broilers were *C. jejuni* and 9.9% were *C*. *coli.*

A total of 80% and 84.4% of the *C. jejuni* and *C. coli* isolates respectively were resistant to ciprofloxacin and nalidixic acid. Travelers to Asia have been shown to carry resistant *Campylobacter* reflecting the above situation [5]. In contrast, Norstorm et al. 2007 [44] demonstrated no resistance to quinolones in *C. jejuni* isolated from broilers in Norway. The reported low resistance has been attributed to low usage of antimicrobials in Norwegian broiler production. Therefore, patterns and practices of antimicrobial usage in food animals can determine the development of antimicrobial resistance in foodborne pathogens such as in *Campylobacter.*

The high prevalence of antimicrobial resistance seen in *Campylobacter* isolates found in this study could be due to misuse of antimicrobials in the broiler industry. Therefore, a detailed survey on the types and pattern of antimicrobial usage in broiler industry will be beneficial in controlling the transmission of antimicrobial resistance between livestock and humans. 

## Figures and Tables

**Table 1:** Prevalence of *Campylobacter* from different sources.

Source		No. Positive/No. Tested	Percentage (%) of Positive Neck Skin and Meat Samples
Semi-automated processing plants (neck skin)	Plant A	19/54	35.1
	Plant B	9/48	18.7
	Total	28/102	27.4
Wet market processing (neck skin)		12/25	48
Retail shops (meat)		22/37	59.4

Simple statistical analysis of the presence of *Campylobacter* in neck skins produced at semi-automated processing plants and wet market processing using Fisher’s exact test resulted in a *p* value equivalent to 0.0570 (*p* = 0.0570), which indicated that there is no difference between semi-automated vs. wet market processing on the contamination of neck skins with *Campylobacter*.

**Table 2:** Presence of *C. jejuni* and *C. coli* among different types of samples.

Category	Sample Type	Number of *Campylobacter* Isolates	Number of *C. jejuni* Isolates	Number of *C. coli* Isolates
Retail shops	Meat	5	1	4
Wet markets	Neck skin	8	0	8
	Caeca	8	2	6
Semi-automated processing	Neck skin	8	4	4
	Caeca	36	13	23
TOTAL		65	20	45
Percentage			30.7%	69.2%

**Table 3:** Percentage of isolates resistant to tested antimicrobials.

Antibiotic	Percentage of Isolates Resistant to the Antimicrobials	
	*C. coli* (*n* = 45)	*C. jejuni* (*n* = 20)
erythromycin	5 (11.11%)	1 (5%)
gentamicin	1 (2.22%)	2 (10%)
streptomycin	2 (4.44%)	1 (05%)
neomycin	4 (8.88%)	2 (10%)
tetracycline	11 (24.44%)	17 (85%)
ciprofloxacin	38 (84.44%)	16 (80%)
nalidixic acid	38 (84.44%)	16 (80%)
tulathromycin	3 (6.66%)	1 (05%)
ampicillin	4 (8.8%)	9 (45%)
claritromycin	3 (6.66%)	0 (0%)
sulphamethoxazole	1 (2.22%)	0 (0%)
chloramphenicol	1 (2.22%)	0 (0%)
